# Supplementary material for: Three-photon in vivo imaging of neurons and glia in the medial prefrontal cortex with sub-cellular resolution
Source: Commun Biol. 2025 May 23;8:795. doi: 10.1038/s42003-025-08079-8 (PMC12102176; doi:10.1038/s42003-025-08079-8)
Supplement: Supplementary file 1 — Supplementary Material [file 42003_2025_8079_MOESM1_ESM.pdf]

Supplementary material

Three-photon in vivo imaging of neurons and glia in the medial prefrontal cortex with sub-cellular resolution

Falko Fuhrmann[1,#], Felix C. Nebeling[1,#], Fabrizio Musacchio[1,#], Manuel Mittag[1], Stefanie Poll[1,7], Monika Müller[1], Eleonora Ambrad Giovannetti[1], Michael Maibach[1], Barbara Schaffran[2], Emily Burnside[2], Ivy Chi Wai Chan[3], Alex Simon Lagurin[3], Nicole Reichenbach[4], Sanjeev Kaushalya[5], Hans Fried[5], Stefan Linden[6], Gabor C. Petzold[4,10], Gaia Tavosanis[3,8,9], Frank Bradke[2], and Martin Fuhrmann[1]*

[#] These authors contributed equally

[1]Neuroimmunology and Imaging Group, German Center for Neurodegenerative Diseases (DZNE), Bonn, Germany

[2]Axon Growth and Regeneration Group, German Center for Neurodegenerative Diseases (DZNE), Bonn, Germany

[3]Dynamics of Neuronal Circuits Group, German Center for Neurodegenerative Diseases (DZNE), Bonn, Germany

[4]Vascular Neurology Group, German Center for Neurodegenerative Diseases (DZNE), Bonn, Germany

[5]Core Research Facilities and Services, Light Microscope Facility, German Center for Neurodegenerative Diseases (DZNE), Bonn, Germany

[6]Department of Physics, Nanophotonics, University of Bonn, Bonn, Germany

[7]IEECR, University Clinic Bonn, Germany

[8]LIMES, University of Bonn, Germany

[9]Department of Developmental Biology, RWTH, Aachen, Germany

[10]Division of Vascular Neurology, University Hospital Bonn, Bonn, Germany

Supplementary Text

In vivo 3P imaging of the spinal cord

Like the corpus callosum, the spinal cord contains many myelinated axons. 3P-imaging in combination with AO significantly improved the penetration depth in comparison to two-photon imaging [1-3]. Our results confirm previous findings and provide evidence that 3P-imaging in the dorsal horn of the spinal cord can be used to acquire subcellular resolution images at 300 µm depth without AO. It should be noted as for hippocampal preparations, that coverslip placement needs to be adjusted in a way that only few myelinated axons directly cross the light path. Imaging through highly scattering layers of myelinated axons degrades the point spread function and can only be partially corrected with AO.

In-vivo imaging of the Drosophila Mushroom body developmental assembly and activity in response to odors, through the intact cuticle

Live imaging of the *Drosophila* brain, combined with recently available whole brain connectomes is a powerful tool to understand the organizational logic of fundamental circuits [4,5]. In *Drosophila*, the mushroom body (MB) is responsible for olfactory associative learning. Its input circuit, the calyx, includes the Kenyon cells (KCs) receiving olfactory information from the projection neurons (PNs) [6].

In adult flies, odor objects are represented by the sparse odor-specific activation patterns of the KCs population [7,8]. The major obstacle to functionally image the MB with high spatial resolution is the light scattering caused by fat bodies and trachea underneath the cuticle, that are thus typically removed. However, this increases the risk of damaging the MB underneath. Only recently successful preparations of adult flies were established leaving the cuticle intact in combination with 3P-imaging [9,10]. While this improvement created a path to longer term imaging, those preparations required partial compression of the fly's head to achieve sufficient depth penetration. It is unclear whether flies survive that procedure for more than 24 hr. Therefore, with the use of 3P, we have developed a reversible mounting procedure **(Supplementary Fig. 6a)** that effectively exposes the cell bodies of KCs from the posterior side **(Supplementary Fig. 6b)**. 3P-imaging at 1300 nm excitation wavelength enabled deeper penetration through the fat body, resolving the small closely-packed KCs cell bodies (3-5 µm in diameter) **(Supplementary Fig. 6c)**. We performed Ca^{2+}-imaging of KCs expressing GCaMP6f and a nuclear marker (NLS-Cherry) in male flies **(Supplementary Fig. 6c).** Flies were head-fixed under the 3P-imaging setup and stimulated with individual odors **(Supplementary Fig. 6d)**. Segmentation of KCs was carried out based on nuclear NLS-mCherry expression **(Supplementary Fig. 6e, Supplementary Video 12)**. Corresponding dF/F_0 of the GCaMP signal was calculated for individual KCs **(Supplementary Fig. 6f)**. KCs that responded to the same odor for all three trials were considered as responding units

(Supplementary Fig. 6g). Here, we provide a refined 3P-imaging approach that does not include head compression, while allowing resolution of individual mushroom body neurons and recording of their Ca^{2+}-transients. This approach will be useful to correlate neuronal activity with long-term behavior experiments in *Drosophila*. Thereby, the toolkit to understand the fly brain will be greatly advanced.

Supplemental Figures

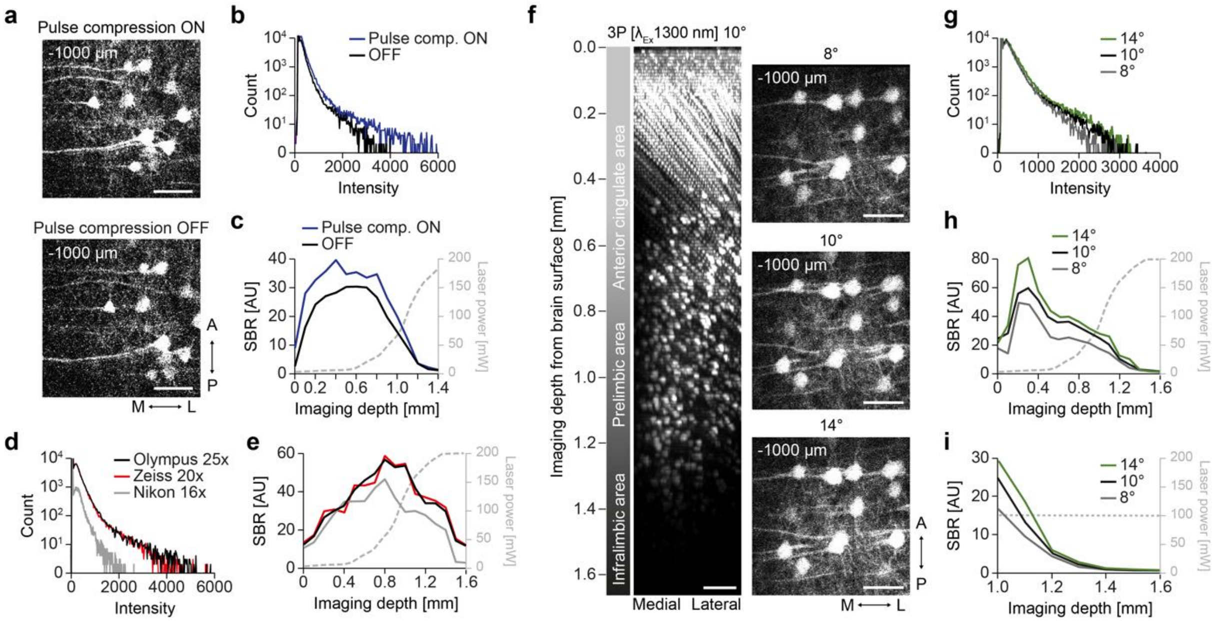

Supplementary Figure 1. Optomechanical adjustments to improve the microscope.

(a) Exemplary images of z-sections acquired at 1 mm depth with pulse compensation ON and OFF. Scale bars: 50 µm.

(b) Comparison of fluorescence histograms at 1 mm depth with or without pulse compression.

(c) SBR as a function of imaging depth for the z-stacks recorded with or without pulse compression including laser-power as a function of imaging depth.

(d) Comparison of fluorescence histograms at 1 mm depth for the three different objectives (Olympus 25x, Zeiss 20x and Nikon 16x)

(e) SBR as a function of imaging depth for the z-stacks recorded with the three different objectives including laser-power as a function of imaging depth.

(f) 3D reconstruction of a z-stack recorded from a depth up to 1600 µm below the brain surface (left). Exemplary images at 1 mm depths (right) for 8°, 10° and 14° modification on the detection optics. Scale bars: 100 µm, 50 µm.

(g) Comparison of fluorescence histograms at 1 mm depth for 8°, 10° and 14° modification on the detection optics.

(h) SBR as a function of imaging depth for the identical z-stacks recorded with 8°, 10° and 14° modification on the detection optics, including laser-power usage as a function of imaging depth.

(i) SBR as a function of imaging depth for the identical z-stacks recorded with 8°, 10° and 14° modification on the detection optics at constant laser-power (100 mW).

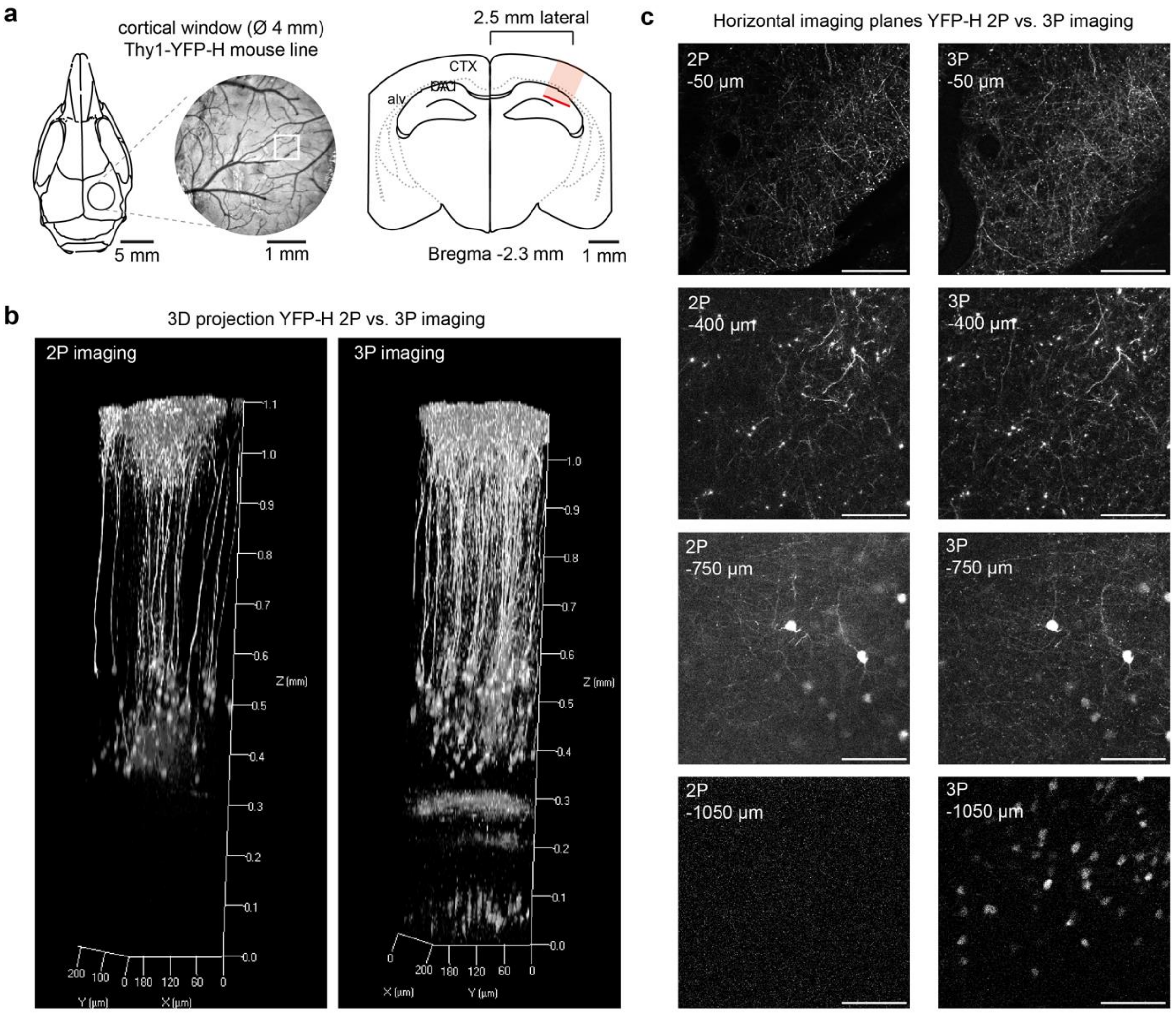

Supplementary Figure 2. 2P- versus 3P-imaging through the cortex into the hippocampus.

(a) Schematic of the cranial window position above the somatosensory cortex (left), and red marked area of the lateral imaging region on a coronal section (right).

(b) 3D reconstruction of a z-stack acquired with 2P (left) versus 3P (right) imaging in a Thy1-YFP-H mouse (4 month old), expressing YFP in a subset of excitatory neurons.

(c) Exemplary images of z-sections acquired at different depth comparing 2P with 3P excitation. Scale bars: 100 µm.

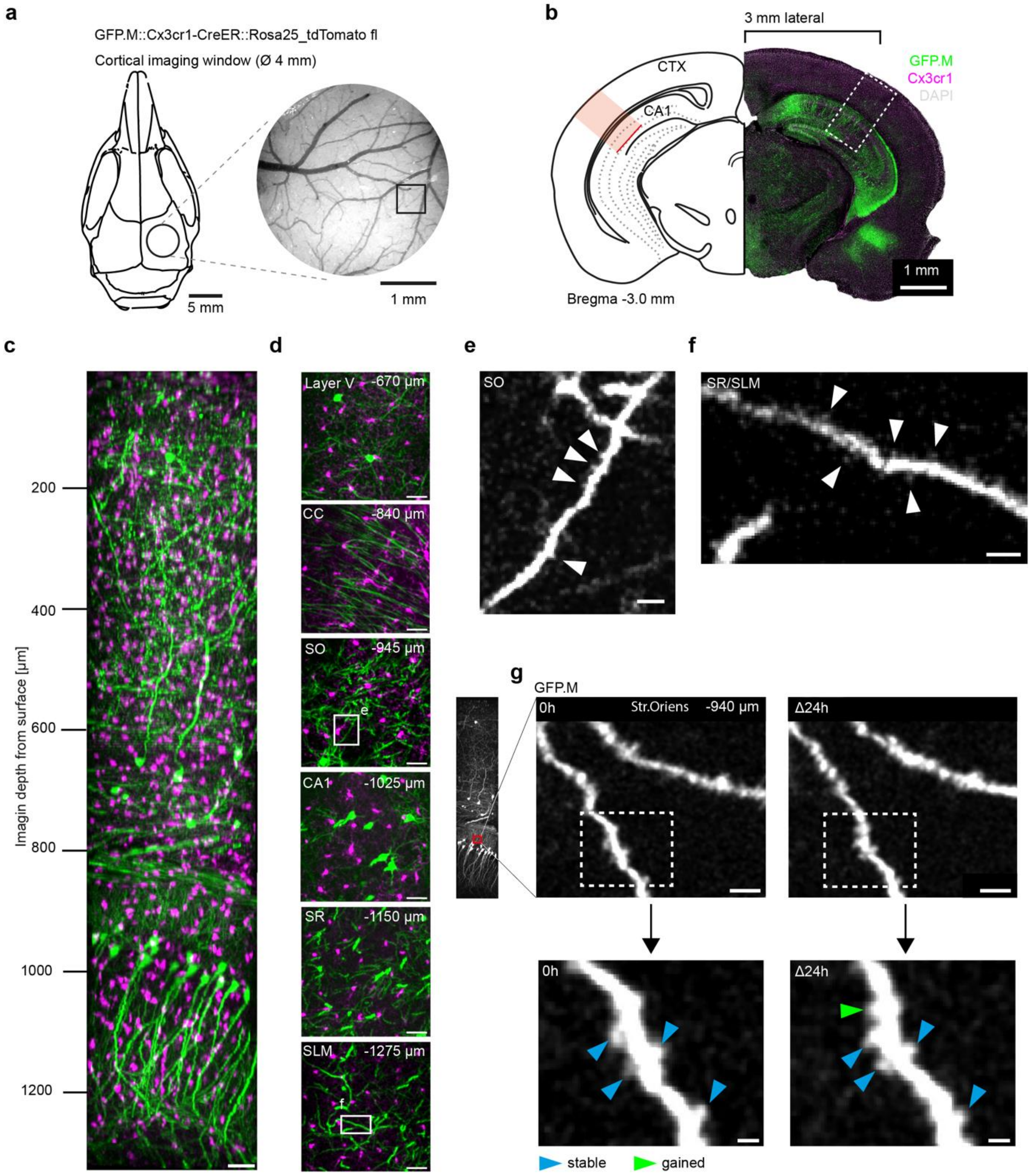

Supplementary Figure 3. 3P-imaging through the cortex into the hippocampus with subcellular resolution.

(a) Schematic of the cranial window position and image of the brain surface and vasculature in a 2.5 months old mouse.

(b) Schematic and coronal section illustrating the lateral imaging position.

(c, d) 3D reconstruction and exemplary images of a z-stack acquired at the ROI marked in (a). Scale bars: 50 µm, 20 µm.

(e) Exemplary image of dendritic spines on CA1 pyramidal neurons in SO. Scale bar: 5 µm.

(f) Exemplary image of dendritic spines on CA1 pyramidal neurons in SR/SLM. Scale bar: 5 µm.

(g) Longitudinal imaging of dendritic spines on CA1 pyramidal neurons in SO. Arrowheads indicate stable (blue), gained (green) spines. Scale bar: 5 µm (upper panel), 2 µm (lower panel).

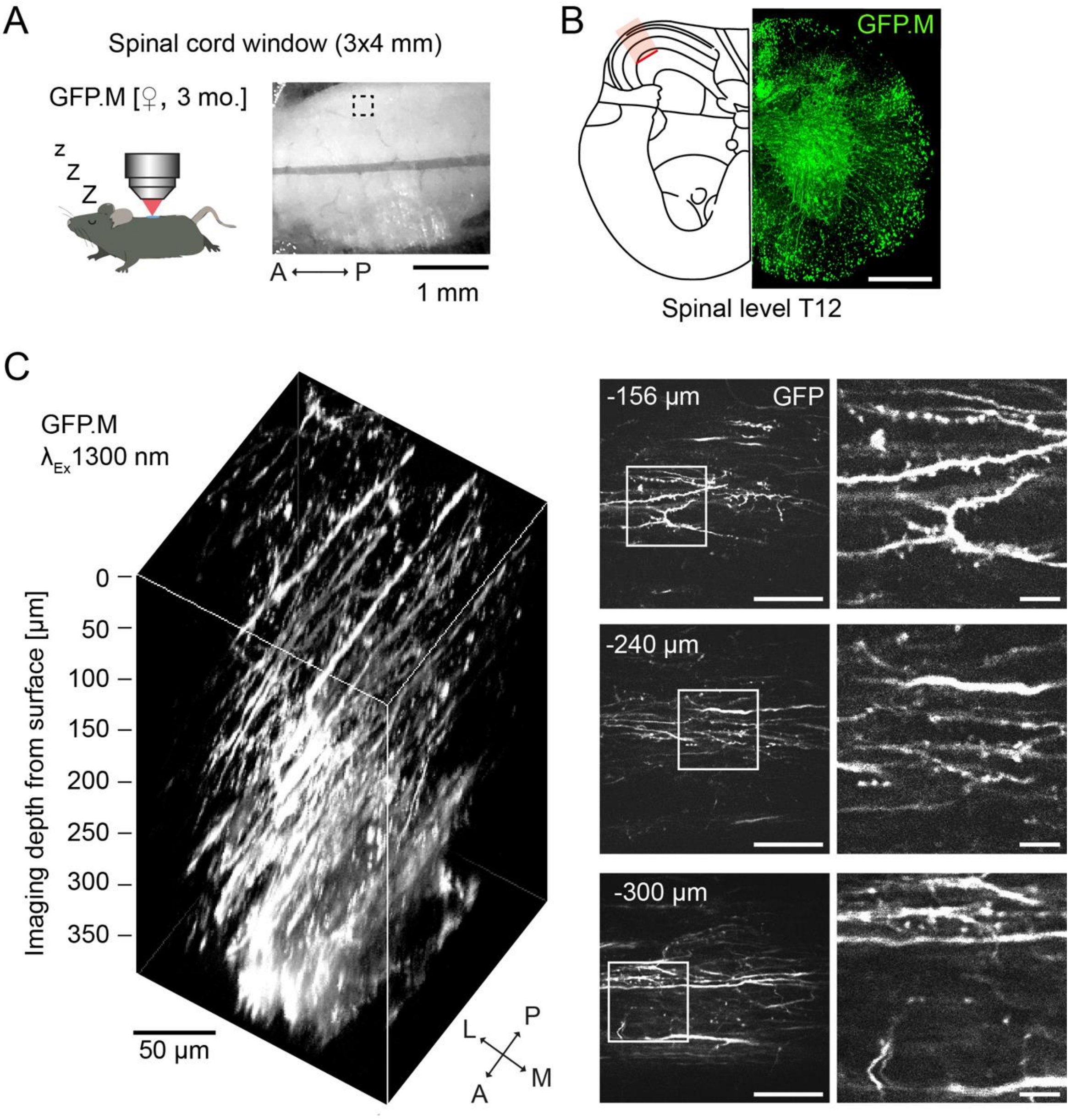

Supplementary Figure 4. In vivo 3P imaging of the spinal cord

(a) Schematic showing spinal cord in vivo imaging in an anesthetized GFP-M mouse and a picture illustrating the view into the implanted spinal cord window with indicated imaging ROI position.

(b) Schematic of a transverse section spinal level T12 (left panel) and a confocal microscopy picture from a corresponding section in a GFP.M transgenic mouse (right panel). Scale bar: 1 mm.

(c) 3D reconstruction of 132 x-y frames from spinal cord surface to 390 µm below taken at a depth increment of 3 µm with 1300 nm Excitation (left) and exemplary individual z-planes recorded at different depths as indicated in the pictures with magnified ROIs (right). Scale bars: 50 µm (middle panel), 10 µm (right panel).

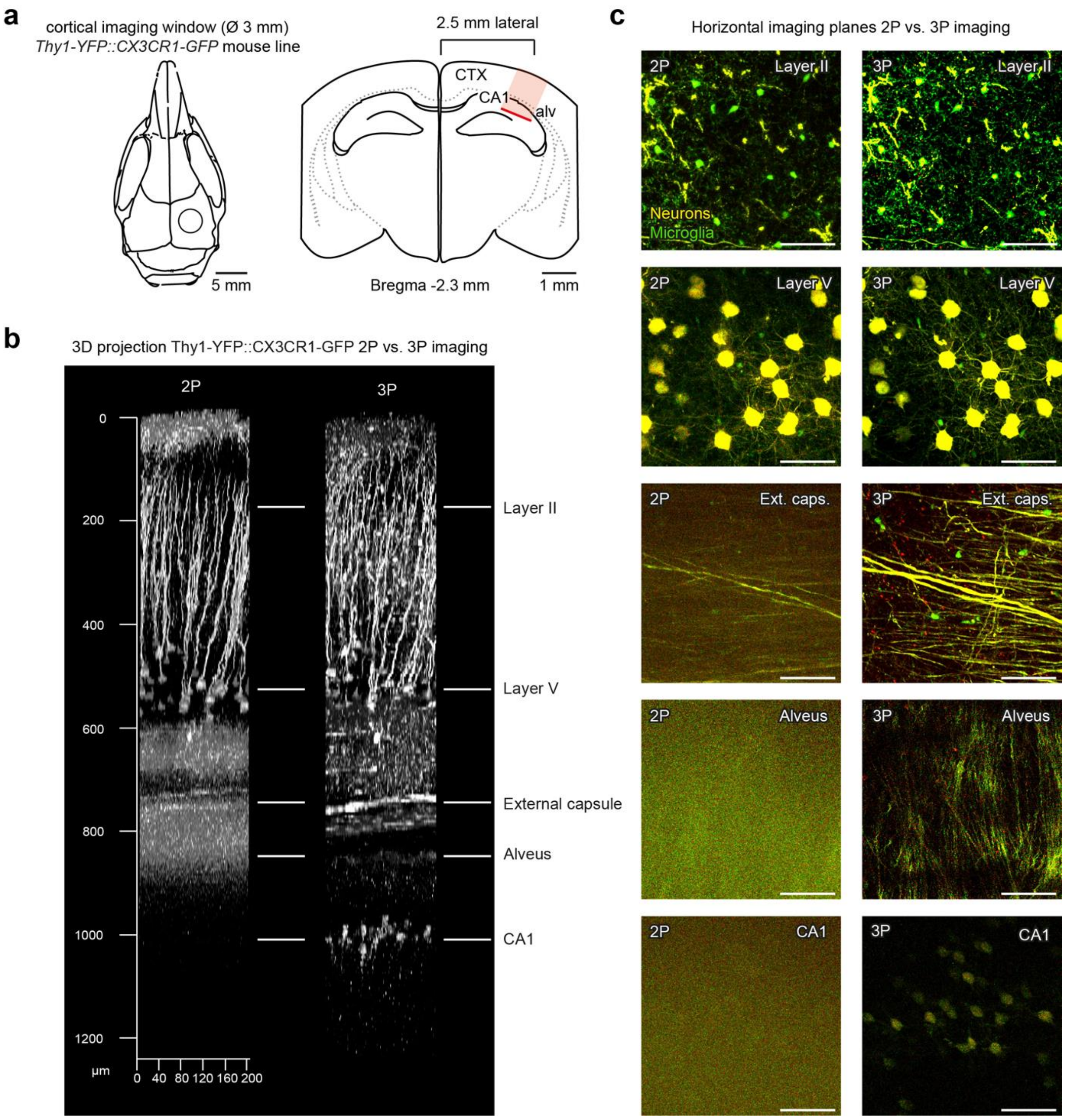

Supplementary Figure 5. 2P- versus 3P-imaging through the cortex into the hippocampus.

(a) Schematic of the cranial window position above the somatosensory cortex (left), and red marked area of the lateral imaging region on a coronal section (right).

(b) 3D reconstruction of a z-stack acquired with 2P (left) versus 3P (right) imaging in a Thy1-YFP-H::Cx3cr1^{GFP} mouse (4 month old).

(c) Exemplary images of z-sections acquired at different depth comparing 2P with 3P excitation. Microglia are labeled in green (GFP) and neurons are labeled in yellow (YFP). Scale bars: 50 µm.

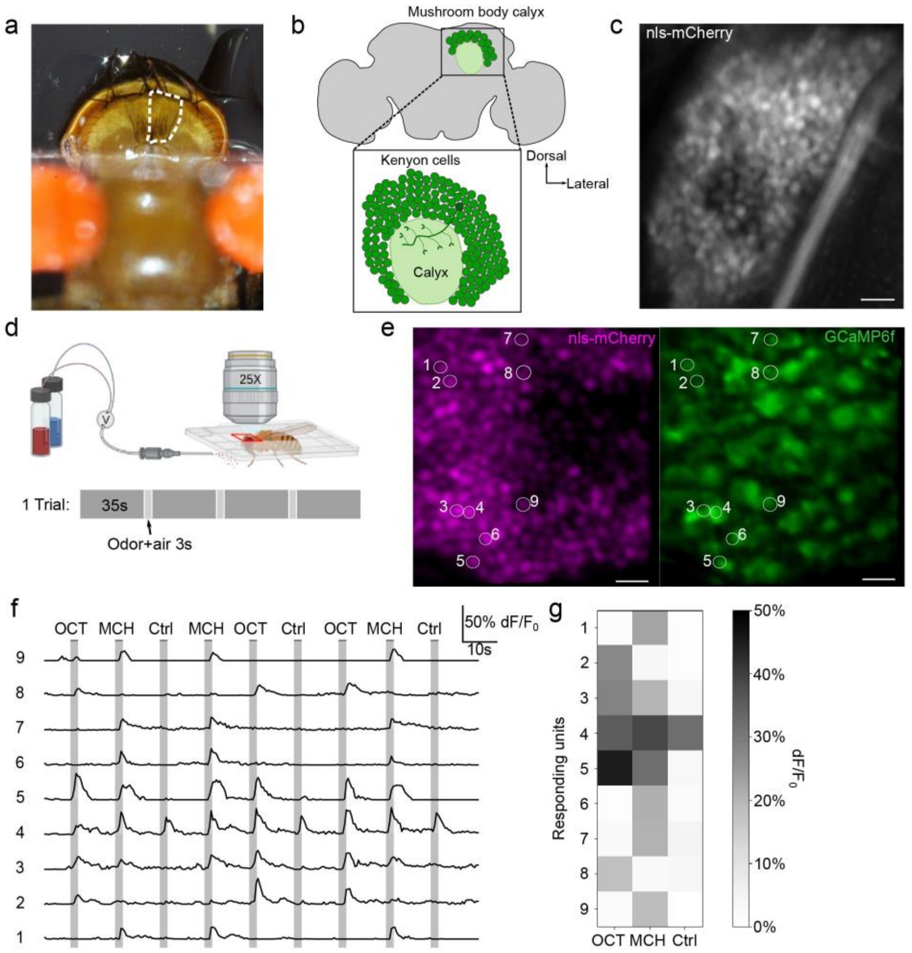

Supplementary Figure 6: *In-vivo* functional imaging of the *Drosophila* Mushroom body with intact cuticle.

(a) Image of a fly attached to a glass coverslip, exposing the posterior part of the head capsule for functional imaging of the KCs (the dotted line region).

(b) Schematics of the adult fly brain, with the highlighted MB calyx. The KCs cell bodies (green circles) surround the MB calyx, where their dendrites receive PN olfactory inputs. The imaging plane (insert) contains around 150 KCs cell bodies across the calyx.

(c) Example volume of the KCs nuclei expressing nls-mCherry (20µm thick with 40 sections). Scale bar: 10µm.

(d) Scheme of the experimental setup and odor stimulation protocol (adapted from Prisco et al., 2021). 3 s of odor delivery of each of the 3 odors (3-Octanol (OCT), 4-Methylcyclohexanol (MCH) and mineral oil (Ctrl)), inter-odor interval of 35 s.

(e) Left panel: KCs nuclei labelled with nls-mCherry. Right panel: Cell bodies of KCs expressing GCaMP6f 1. KCs were recorded 16 µm below the cuticle .The activity of each circled responding unit is shown in (f). Scale bar: 10 µm.

(f) Time course of response (dF/F_0) of the responding units to a series of odor stimuli. The numbers correspond to the circles highlighted in (e).

(g) Trial average peak response of the responding units.

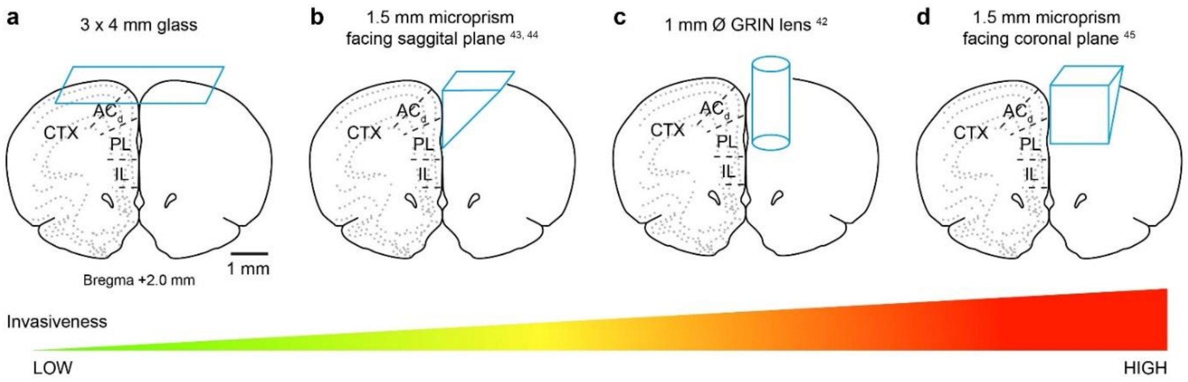

Supplementary Figure 7. Different technical approaches to access mPFC with in vivo imaging

(a) Schematic of a coronal section 2mm anterior of Bregma illustrating the 3x4mm glass position for imaging access to both hemispheres, all mPFC areas and all mPFC layers in a single preparation.

(b) Schematic of a 1.5mm microprism implanted into the fissure facing the sagittal plane. Restricted imaging access in a single preparation to superficial mPFC areas and superficial mPFC layers.

(b) Schematic of an implanted 1mm Ø GRIN lens into the mPFC. Imaging access in a single preparation is restricted to one mPFC area with several mPFC layers.

(d) Schematic of an implanted 1.5mm microprism facing the coronal plane. Restricted imaging access in a single preparation to superficial mPFC areas but all mPFC layers.

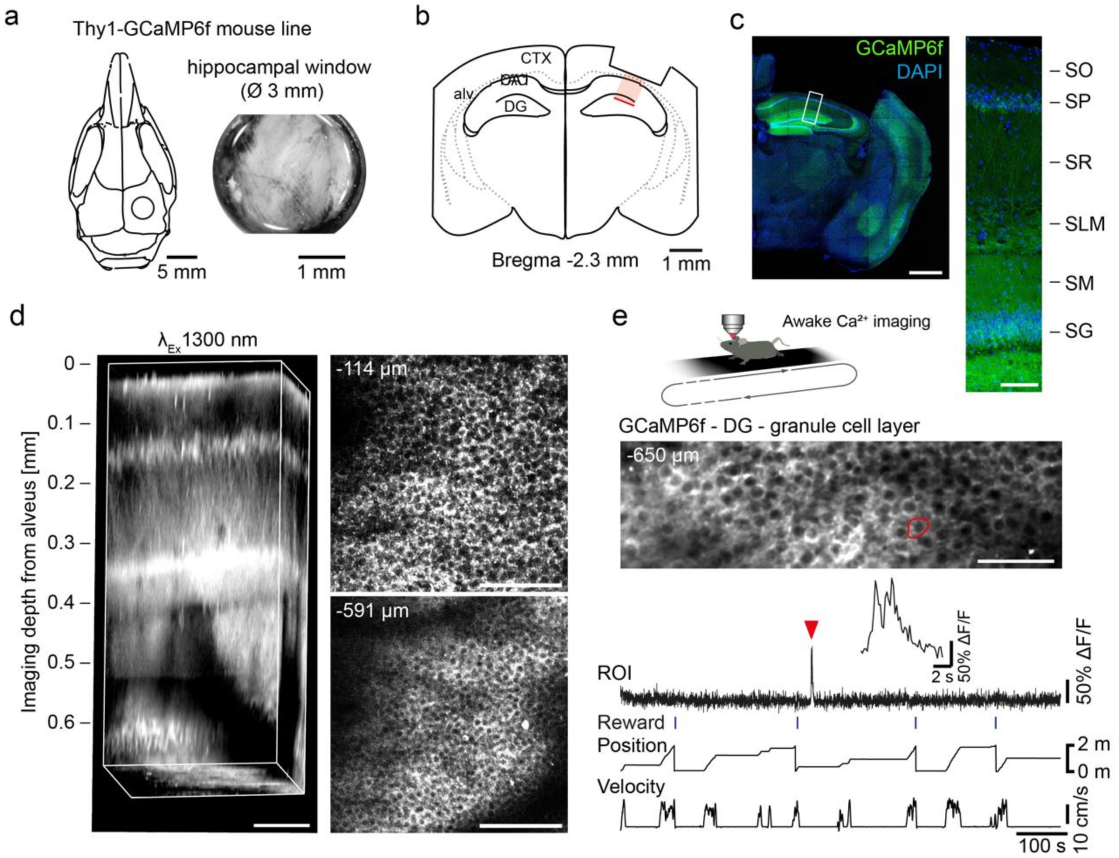

Supplementary Figure 8. Dentate gyrus functional three-photon Ca^{2+}-imaging.

(a) Schematic of the hippocampal window placement in a Thy1-GCaMP6f mouse.

(b) Schematic showing the implantation side of the hippocampal window in the right hemisphere and the imaging region in red.

(c) Coronal section 2.3 mm anterior bregma with expression of GCaMP6f (green) and DAPI staining (blue). The right panel shows a zoom of the box in the left panel labelling the different dorsal hippocampal layers. Stratum oriens (SO), pyramidale (SP), radiatum (SR), lacunosum moleculare (SLM), moleculare (SM), granulare (SG). Scale bars: 1 mm, 100 µm.

(d) 3D reconstruction of an imaging volume spanning from SO to SG up to 700 µm deep into the dorsal hippocampus (left panel). Exemplary images of the pyramidal and granule cell layer at 140 and 680 µm depth respectively (right panel). Scale bars: 100 µm.

(e) Schematic of awake head-fixed Ca^{2+}-imaging of a mouse running on a linear belt treadmill (upper panel). Example image of granule cells in the dentate gyrus with one ROI (2nd panel) of an active neuron. 20-minute Ca^{2+}-recording of the selected neuron and zoom of the single measured Ca^{2+}-transient (3rd panel). The lower two panels show the position of the mouse on the belt and its velocity (last two panels). Scale bar: 50 µm.

Supplementary Data 1.
Contains the source data for all graphs in the paper.

Supplementary Video 1.
In-vivo 3P z-scan of 320 x-y frames from brain surface to 1600µm below, taken at a depth increment of 5µm in the mPFC of YFP-H transgenic mouse.

Supplementary Video 2.
In-vivo 2P mPFC z-scan with 920nm excitation in a YFP-H transgenic mouse.

Supplementary Video 3.
In-vivo 3P mPFC z-scan with 1300nm excitation in a YFP-H transgenic mouse.

Supplementary Video 4.
In-vivo 3P Cortex to Hippocampus z-scan of 265 x-y frames from surface to 1325 µm below taken at a depth increment of 5 µm with 1300nm excitation in a GFP.M::Cx3cr1-CreER::Rosa25_tdTomato transgenic mouse.

Supplementary Video 5.
In-vivo 3P z-scan of 132 x-y frames from spinal cord surface to 390 µm below taken at a depth increment of 3 µm with 1300 nm excitation in a Thy1-GFP-M transgenic mouse.

Supplementary Video 6.
In-vivo 3P mPFC z-scan with 1300nm excitation in a Thy1-GFP-M transgenic mouse.

Supplementary Video 7.
In-vivo 3P mPFC z-scan with 1650nm excitation in a Cx3Cr1-creER2 Rosa tdTomato mouse on day0.

Supplementary Video 8.
In-vivo 3P mPFC z-scan with 1650nm excitation in a Cx3Cr1-creER2 Rosa tdTomato mouse on day1.

Supplementary Video 9.
Microglial fine process motility in the mPFC at d0 and d1.

Supplementary Video 10.

In-vivo 3P z-scan of 406 x-y frames from brain surface to 1200 µm below, acquired at a depth increment of 3 µm in the mPFC of a GLAST-CreERT2::GCaMP5g::tdTomato transgenic mouse.

Supplementary Video 11.

In vivo 3P recording of GCaMP5g-positive astrocytes at 1000 µm below surface. GCaMP (green channel), tdTomato (magenta channel), merge.

Supplementary Video 12.

In-vivo 3P functional imaging of the *Drosophila* Mushroom body with intact cuticle.

Supplementary Video 13.

In-vivo 3P z-scan from brain surface to 1420µm below in a vGlut2-Cre mouse expressing GCaMP6s in glutamatergic neurons in the mPFC.

Supplementary Video 14.

In-vivo 3P recording of GCaMP6s-positive glutamatergic neurons in the mPFC at a depth of 1100 µm.

Supplementary Video 15.

In-vivo 3P z-scan from SO to SG up to 700 µm deep into the dorsal hippocampus through a hippocampal window in a Thy1-GCaMP6f transgenic mouse.

Supplementary References

1 Rodríguez, C. *et al.* An adaptive optics module for deep tissue multiphoton imaging in vivo. *Nat Methods* **18**, 1259-1264, doi:10.1038/s41592-021-01279-0 (2021).

2 Schaffran, B., Hilton, B. J. & Bradke, F. Imaging in vivo dynamics of sensory axon responses to CNS injury. *Exp Neurol* **317**, 110-118, doi:10.1016/j.expneurol.2019.02.010 (2019).

3 Borjini, N., Paouri, E., Tognatta, R., Akassoglou, K. & Davalos, D. Imaging the dynamic interactions between immune cells and the neurovascular interface in the spinal cord. *Exp Neurol* **322**, 113046, doi:10.1016/j.expneurol.2019.113046 (2019).

4 Hulse, B. K. *et al.* A connectome of the Drosophila central complex reveals network motifs suitable for flexible navigation and context-dependent action selection. *Elife* **10**, doi:10.7554/eLife.66039 (2021).

5 Li, F. *et al.* The connectome of the adult Drosophila mushroom body provides insights into function. *Elife* **9**, doi:10.7554/eLife.62576 (2020).

6 Modi, M. N., Shuai, Y. & Turner, G. C. The Drosophila Mushroom Body: From Architecture to Algorithm in a Learning Circuit. *Annu Rev Neurosci* **43**, 465-484, doi:10.1146/annurev-neuro-080317-0621333 (2020).

7 Endo, K. & Kazama, H. Central organization of a high-dimensional odor space. *Current opinion in neurobiology* **73**, 102528, doi:10.1016/j.conb.2022.102528 (2022).

8 Gruntman, E. & Turner, G. C. Integration of the olfactory code across dendritic claws of single mushroom body neurons. *Nat Neurosci* **16**, 1821-1829, doi:10.1038/nn.3547 (2013).

9 Aragon, M. J. *et al.* Multiphoton imaging of neural structure and activity in Drosophila through the intact cuticle. *Elife* **11**, doi:10.7554/eLife.69094 (2022).

10 Tao, X. *et al.* Transcutical imaging with cellular and subcellular resolution. *Biomed Opt Express* **8**, 1277-1289, doi:10.1364/boe.8.001277 (2017).
