# Supplementary material for: Three-photon in vivo imaging of neurons and glia in the medial prefrontal cortex with sub-cellular resolution
Source: Commun Biol. 2025 May 23;8:795. doi: 10.1038/s42003-025-08079-8 (PMC12102176; doi:10.1038/s42003-025-08079-8)
Supplement: Supplementary file 2 — Description of Additional Supplementary Files [file 42003_2025_8079_MOESM2_ESM.docx]

Description of Additional Supplementary Files

**File name:** Supplementary Data 1

**Description:** Contains the source data for all graphs in the paper.

**File name:** Supplementary Video 1

**Description:** In-vivo 3P z-scan of 320 x-y frames from brain surface to 1600µm below, taken at a depth increment of 5µm in the mPFC of YFP-H transgenic mouse.

**File name:** Supplementary Video 2

**Description:** In-vivo 2P mPFC z-scan with 920nm excitation in a YFP-H transgenic mouse.

**File name:** Supplementary Video 3

**Description:** In-vivo 3P mPFC z-scan with 1300nm excitation in a YFP-H transgenic mouse.

**File name:** Supplementary Video 4

**Description:** In-vivo 3P Cortex to Hippocampus z-scan of 265 x-y frames from surface to 1325 µm below taken at a depth increment of 5 µm with 1300nm excitation in a GFP.M::Cx3cr1- CreER::Rosa25_tdTomato transgenic mouse.

**File name:** Supplementary Video 5

**Description:** In-vivo 3P z-scan of 132 x-y frames from spinal cord surface to 390 µm below taken at a depth increment of 3 µm with 1300 nm excitation in a Thy1-GFP-M transgenic mouse.

**File name:** Supplementary Video 6

**Description:** In-vivo 3P mPFC z-scan with 1300nm excitation in a Thy1-GFP-M transgenic mouse.

**File name:** Supplementary Video 7

**Description:** In-vivo 3P mPFC z-scan with 1650nm excitation in a Cx3Cr1-creER2 Rosa tdTomato mouse on day0.

**File name:** Supplementary Video 8

**Description:** In-vivo 3P mPFC z-scan with 1650nm excitation in a Cx3Cr1-creER2 Rosa tdTomato mouse on day1.

**File name:** Supplementary Video 9

**Description:** Microglial fine process motility in the mPFC at d0 and d1.

**File name:** Supplementary Video 10

**Description:** In-vivo 3P z-scan of 406 x-y frames from brain surface to 1200 µm below, acquired at a depth increment of 3 µm in the mPFC of a GLASTCreERT2::GCaMP5g::tdTomato transgenic mouse.

**File name:** Supplementary Video 11

**Description:** In vivo 3P recording of GCaMP5g-positive astrocytes at 1000 µm below surface. GCaMP (green channel), tdTomato (magenta channel), merge.

**File name:** Supplementary Video 12

**Description:** In-vivo 3P functional imaging of the Drosophila Mushroom body with intact cuticle.

**File name:** Supplementary Video 13

**Description:** In-vivo 3P z-scan from brain surface to 1420µm below in a vGlut2-Cre mouse expressing GCaMP6s in glutamatergic neurons in the mPFC.

**File name:** Supplementary Video 14

**Description:** In-vivo 3P recording of GCaMP6s-positive glutamatergic neurons in the mPFC at a depth of 1100 µm.

**File name:** Supplementary Video 15

**Description:** In-vivo 3P z-scan from SO to SG up to 700 µm deep into the dorsal hippocampus through a hippocampal window in a Thy1-GCaMP6f transgenic mouse.
