# Supplementary material for: Three-photon in vivo imaging of neurons and glia in the medial prefrontal cortex with sub-cellular resolution
Source: Commun Biol. 2025 May 23;8:795. doi: 10.1038/s42003-025-08079-8 (PMC12102176; doi:10.1038/s42003-025-08079-8)
Supplement: Supplementary file 19 — Reporting summary [file 42003_2025_8079_MOESM19_ESM.pdf]

nature portfolio

Corresponding author(s): Martin Fuhrmann

Last updated by author(s): Mar 27, 2025

Reporting Summary

Nature Portfolio wishes to improve the reproducibility of the work that we publish. This form provides structure for consistency and transparency in reporting. For further information on Nature Portfolio policies, see our Editorial Policies and the Editorial Policy Checklist.

Statistics

For all statistical analyses, confirm that the following items are present in the figure legend, table legend, main text, or Methods section.

n/a	Confirmed	
☐	☒	The exact sample size (n) for each experimental group/condition, given as a discrete number and unit of measurement
☐	☒	A statement on whether measurements were taken from distinct samples or whether the same sample was measured repeatedly
☐	☒	The statistical test(s) used AND whether they are one- or two-sided<br>*Only common tests should be described solely by name; describe more complex techniques in the Methods section.*
☒	☐	A description of all covariates tested
☐	☒	A description of any assumptions or corrections, such as tests of normality and adjustment for multiple comparisons
☐	☒	A full description of the statistical parameters including central tendency (e.g. means) or other basic estimates (e.g. regression coefficient) AND variation (e.g. standard deviation) or associated estimates of uncertainty (e.g. confidence intervals)
☐	☒	For null hypothesis testing, the test statistic (e.g. F, t, r) with confidence intervals, effect sizes, degrees of freedom and P value noted<br>*Give P values as exact values whenever suitable.*
☒	☐	For Bayesian analysis, information on the choice of priors and Markov chain Monte Carlo settings
☒	☐	For hierarchical and complex designs, identification of the appropriate level for tests and full reporting of outcomes
☒	☐	Estimates of effect sizes (e.g. Cohen's d, Pearson's r), indicating how they were calculated

Our web collection on statistics for biologists contains articles on many of the points above.

Software and code

Policy information about availability of computer code

Data collection	thorimage, Zeiss ZEN blue
Data analysis	imageJ, Imaris 9.6, taro tools, CalmAn, graph pad prism, custom python algorithms for assessing microglia fine procesces motility (MotilA; https://github.com/FabrizioMusacchio/MotilA) and detection and classification of flourescence peaks over time in astrocytic Ca^{2+} imaging (published and referenced in Mat&Meth).

For manuscripts utilizing custom algorithms or software that are central to the research but not yet described in published literature, software must be made available to editors and reviewers. We strongly encourage code deposition in a community repository (e.g. GitHub). See the Nature Portfolio guidelines for submitting code & software for further information.

Data

Policy information about availability of data

All manuscripts must include a data availability statement. This statement should provide the following information, where applicable:

- Accession codes, unique identifiers, or web links for publicly available datasets
- A description of any restrictions on data availability
- For clinical datasets or third party data, please ensure that the statement adheres to our policy

The authors declare that the data supporting the findings of this study are available within the paper, the methods section, and Extended Data files. Raw data are available from the corresponding author upon reasonable request and from DRYAD.

Research involving human participants, their data, or biological material

Policy information about studies with human participants or human data. See also policy information about sex, gender (identity/presentation), and sexual orientation and race, ethnicity and racism.

Reporting on sex and gender	N.A.
Reporting on race, ethnicity, or other socially relevant groupings	N.A.
Population characteristics	N.A.
Recruitment	N.A.
Ethics oversight	N.A.

Note that full information on the approval of the study protocol must also be provided in the manuscript.

Field-specific reporting

Please select the one below that is the best fit for your research. If you are not sure, read the appropriate sections before making your selection.

☒ Life sciences ☐ Behavioural & social sciences ☐ Ecological, evolutionary & environmental sciences

For a reference copy of the document with all sections, see nature.com/documents/nr-reporting-summary-flat.pdf

Life sciences study design

All studies must disclose on these points even when the disclosure is negative.

Sample size	We did not apply statistical methods to predetermine sample sizes, but our chosen sample sizes are similar to those generally employed in the field.
Data exclusions	We did not exclude any data from the experiments.
Replication	All data presented in main figures were successfully replicated. For some supplementary figures, experiments were just carried out one time as a proof of concept or to confirm already published findings. This is stated in the manuscript
Randomization	Mice of both sex were randomly distributed into the experimental groups.
Blinding	All investigators were blinded to group allocation during data analysis.

Reporting for specific materials, systems and methods

We require information from authors about some types of materials, experimental systems and methods used in many studies. Here, indicate whether each material, system or method listed is relevant to your study. If you are not sure if a list item applies to your research, read the appropriate section before selecting a response.

Materials & experimental systems

n/a	Involved in the study	
☒	☐	Antibodies
☒	☐	Eukaryotic cell lines
☒	☐	Palaeontology and archaeology
☐	☒	Animals and other organisms
☒	☐	Clinical data
☒	☐	Dual use research of concern
☒	☐	Plants

Methods

n/a	Involved in the study	
☒	☐	ChIP-seq
☒	☐	Flow cytometry
☒	☐	MRI-based neuroimaging

Animals and other research organisms

Policy information about studies involving animals; ARRIVE guidelines recommended for reporting animal research, and Sex and Gender in Research

Laboratory animals	mus musculus, C57BL6, adult mice older than 2 months
Wild animals	N.A.
Reporting on sex	mice of both sexes were used for the experiments.
Field-collected samples	N.A.
Ethics oversight	Landesamt für Natur, Umwelt und Verbraucherschutz (LANUV)" of North-Rhine Westphalia (Office for Nature, Environment and Consumer Protection of North Rhine-Westphalia) in Recklinghausen, Germany, under the licenses 81-02.04.2019.A084; 81-02.04.2018.A239; 81-02.04.2020.A059; 84-02.04.2017.A098

Note that full information on the approval of the study protocol must also be provided in the manuscript.

Plants

Seed stocks	N.A.
Novel plant genotypes	N.A.
Authentication	N.A.
